# NLRP3 inflammasome-a likely target for the treatment of immunologic conjunctivitis: A protocol for systematic review and meta-analysis

**DOI:** 10.1371/journal.pone.0296994

**Published:** 2024-01-26

**Authors:** Ruoxi Liu, Yi Fang, Fang Yang, Donghui Liu

**Affiliations:** 1 Heilongjiang University of Chinese Medicine, Harbin, Heilongjiang, China; 2 Heilongjiang Mingshui Kangying Hospital, Suihua, Heilongjiang, China; 3 The EYE Hospital of Daqing, Daqing, Heilongjiang, China; 4 Senhai Hospital, Harbin, Heilongjiang, China; University of New South Wales, AUSTRALIA

## Abstract

**Background:**

Immune-mediated conjunctivitis is a prevalent ocular ailment characterized by inflammation and immune reactions in the conjunctiva. However, the precise causes and therapeutic approaches for this condition remain the main focus for numerous ophthalmological specialists. Recently, accumulating evidence from human and mouse experiments has demonstrated the critical involvement of the NLRP3 inflammasome, IL-1β, and IL-18 in the development of allergic diseases. Targeting specific NLRP3 inflammasome and its related inhibitors may hold potential as therapeutic agents for immunologic conjunctivitis. Despite this, there has been no systematic review specifically addressing the treatment of immunologic conjunctivitis related to NLRP3. Therefore, this study aims to conduct a systematic review and meta-analysis of currently published randomized controlled trials (RCTs) on NLRP3-related treatments for immunologic conjunctivitis patients, with the goal of evaluating their efficacy and safety.

**Methods:**

We will conduct a comprehensive search for relevant studies on NLRP3 inflammasome inhibitors or NLRP3-related treatments for immunologic conjunctivitis in various databases including PubMed, EMBASE, Cochrane Library, China National Knowledge Infrastructure (CNKI), VIP, and Wanfang. The search will encompass studies from their respective inception dates to July 2023. A meta-analysis will be performed using data extracted from eligible randomized controlled trials (RCTs), focusing on the clinical manifestations of immunologic conjunctivitis, levels of NLRP3-related factors in serum or tear samples, quality of life outcomes, and adverse events. Review Manager 5.4.1 software will be employed for the meta-analysis, and the results will be analyzed using either random-effects or fixed-effects models, depending on the presence of heterogeneity. The reliability and quality of evidence will be evaluated using the Grading of Recommendations, Development, and Evaluation (GRADE) system.

**Results:**

The findings of this study will yield robust and high-quality evidence regarding the efficacy and safety of NLRP3-related treatments for immunologic conjunctivitis. This evidence will contribute significantly to our understanding of the potential benefits and risks associated with such treatments and will assist healthcare professionals in making informed decisions regarding the management of immunologic conjunctivitis.

**Conclusion:**

This study represents the first comprehensive meta-analysis aiming to evaluate the efficacy and safety of NLRP3-related treatments for immunologic conjunctivitis. The findings from this study will provide valuable evidence to guide clinical management strategies for this disease. The results are anticipated to significantly contribute to the understanding of the therapeutic potential and safety profile of NLRP3-related treatments, offering valuable insights for healthcare professionals involved in the care of patients with immunologic conjunctivitis.

**Trial registration:**

**Systematic review registration:** PROSPERO with registration number CRD42023437076.

## Introduction

Allergic diseases, including asthma, rhinitis, dermatitis, conjunctivitis, and anaphylaxis, have a significant impact on global health, affecting approximately one-third of the general population. These conditions present major challenges in modern medicine [1, 2]. Immunologic conjunctivitis, also known as allergic conjunctivitis, is characterized by a hypersensitive immune response of the conjunctiva to external allergens [1–4]. It encompasses various forms such as spring keratoconjunctivitis, allergic conjunctivitis, seasonal allergic conjunctivitis, perennial allergic conjunctivitis, giant papillary conjunctivitis, and others [5, 6]. Statistics reveal that approximately 15–20% of the population in Europe and the United States suffers from immunologic conjunctivitis annually, with allergic reactions affecting 20%-30% of the U.S. population, half of which can be attributed to immunologic conjunctivitis [1–4]. In China, the central region has the highest prevalence of immunologic conjunctivitis, reaching up to 45.1% [5]. Therefore, it is essential to conduct in-depth research on the pathogenesis of immunologic conjunctivitis and establish effective preventive and treatment strategies.

The prevalent ocular condition of immunologic conjunctivitis is characterized by inflammation and immune reactions in the delicate and transparent conjunctiva, which plays a crucial role in protecting the ocular surface from external irritants. However, when the immune system dysfunctions, the conjunctiva becomes susceptible, leading to inflammation and discomfort. The conjunctiva is regularly exposed to environmental allergens such as pollen, dust mites, animal dander, and is also susceptible to bacterial or other microbial infections. It can also exhibit allergic reactions to medications and food [1, 6, 7]. While immunologic conjunctivitis is generally a benign condition, severe cases can cause visual impairment and significantly impact the patient’s quality of life. Common symptoms include intense itching, tearing, and photophobia, accompanied by signs such as eyelid conjunctiva papillary hypertrophy, conjunctiva congestion, edema, and increased secretion. It is a frequently encountered eye disorder with seasonal and periodic occurrences, often observed in spring and summer, as well as during seasonal transitions in summer and autumn. Recurrence rates are high, and treatment can be challenging [2, 6].

In recent years, significant advancements have been made in understanding immune-mediated conjunctivitis and its diagnostic approaches [1, 8]. However, the precise causes and therapeutic approaches for this condition remain areas of focus for numerous ophthalmological specialists. In terms of treatment, the management of immune-mediated conjunctivitis aims to reduce inflammation, alleviate symptoms, and minimize side effects. Current therapeutic options include the use of topical corticosteroids, immunomodulatory agents, and targeted biologic therapies [8]. Nonetheless, optimizing treatment strategies and achieving long-term remission in refractory cases continue to present ongoing challenges.

The NLRP3 inflammasome has been extensively studied for its activation pathways and pathogenic mechanisms in recent publications in Nature and Science, as it plays a pivotal role in pathogen infections, autoimmune inflammatory reactions, neurodegenerative diseases, cancer, type 2 diabetes, and other conditions [9–11]. Recently, accumulating evidence from human and mouse experiments has demonstrated the outstanding potential of NLRP3-related treatments, including NLRP3 inflammasome inhibitors, Interleukin-1 beta, Interleukin-18, and Caspase-1, in treating immunologic conjunctivitis [12–19]. Specific NLRP3 inflammasome and its related inhibitors may serve as potential therapeutic agents for immunologic conjunctivitis [12–19]. Therefore, in this study, we aim to collect clinical evidence of NLRP3-related treatments for immunologic conjunctivitis and conduct a systematic review and meta-analysis to evaluate their efficacy and safety. By integrating the results from various randomized clinical trials (RCTs), this study will provide valuable insights for clinical decision-making in the treatment of immunologic conjunctivitis.

## Methods

### Study registration and ethics

A protocol, incorporating a detailed search strategy and data analysis method, has been registered with the International Prospective Register of Systematic Reviews (PROSPERO) under the registration number CRD42023437076. The conduct and reporting of this protocol will adhere to the guidelines outlined in the Cochrane Handbook for Systematic Reviews of Interventions and the Preferred Reporting Items for Systematic Review and Meta-Analysis (The PRISMA 2020 statement) checklist [20]. The data for this study will be obtained from published literature, and ethical approval is not required for this systematic review.

### Inclusion and exclusion criteria

#### Types of studies

We will include randomized controlled trials (RCTs) investigating NLRP3-related treatments for immunologic conjunctivitis. Non-clinical trials, non-case-control studies, non-RCTs, and quasi-RCTs will be excluded from the analysis.

#### Participants

We will include studies involving patients diagnosed with immunologic conjunctivitis based on recognized diagnostic criteria. Specifically, as outlined in the "Expert Consensus on the Clinical Diagnosis and Treatment of Immunologic Conjunctivitis" [21, 22] and the "8th Edition of Ophthalmology" [23], immunologic conjunctivitis can be diagnosed when patients present with itching or a foreign body sensation, in conjunction with any one or a combination of the following five criteria: increased papillae and follicles on the conjunctiva, conjunctival congestion, chemosis of the bulbar conjunctiva or eyelid swelling, increased conjunctival sac secretions or meibomian gland orifice secretions, and specific corneal changes. There will be no restrictions on age, race, sex, or profession of the included patients.

#### Interventions

The development stage and specific formulations of interventions targeting NLRP3-related treatments can vary significantly, and there may be distinct considerations associated with each stage such as Clinical Trials, and Approved Treatments. Currently, there is no standardized protocol for dosage, form, or frequency. As a result, this study’s intervention will encompass NLRP3-related treatments without imposing specific restrictions on dosage, form, or frequency.

Furthermore, this study includes various forms and routes of administration for NLRP3-related treatments, such as oral medications, injections, transdermal patches, and other administration methods. NLRP3-related treatments encompass a range of options, including NLRP3 or NLRP3 inflammasome inhibitors (such as small molecules like MCC950), Interleukin-1 beta (IL-1β) treatment (involving anti-IL-1β medications like Anakinra or Canakinumab), Interleukin-18 (IL-18) treatments (involving anti-IL-18 medications), and Caspase-1 treatments (utilizing Caspase-1 inhibitors).

Regarding control treatments, any intervention can be considered, except for NLRP3-related treatments. If a placebo control is employed, it will involve a substance or intervention that is not known to have immunologic effects on conjunctivitis. In cases where a no-treatment control is used, ethical justifiability will be taken into account, as well as the nature and severity of the condition.

#### Outcome indicators

The primary outcomes of this study will encompass the overall effectiveness of treatment, indicated by the total effective rate post-treatment, as well as the levels of NLRP3-related factors in the serum or tears of patients with immunologic conjunctivitis before and after treatment. An effectiveness rate of 60% or higher is deemed effective, 80% or higher is considered highly effective, while rates below 60% are regarded as ineffective. Secondary outcome measures will involve clinical indicators of immunologic conjunctivitis, including papillary and follicular changes, degree of itching, and recurrence rate. Moreover, self-report health-related quality of life questionnaires will be utilized as tertiary outcome measures. Patients recorded their symptoms daily on a scale from 0 to 3, where 0 indicated the absence of symptoms, 1 represented the presence of symptoms without discomfort, 2 signified some discomfort, and 3 denoted marked discomfort. This scoring system was employed to assess discomforted symptoms of immunologic conjunctivitis such as eyelid swelling, itchy eyes, and overall symptoms. The self-report health-related quality of life questionnaires can provide insight into patients’ subjective experiences when evaluating immunologic conjunctivitis [15, 24, 25]. The study will also encompass safety assessments, including the monitoring of adverse events and treatment-related complications, to evaluate the safety profile of the interventions under investigation.

#### Study selection

We will conduct a comprehensive search for pertinent studies on NLRP3 inflammasome inhibitors or NLRP3-related treatments for immunologic conjunctivitis across various databases, including PubMed, EMBASE, CENTRAL, China National Knowledge Infrastructure (CNKI), VIP, and Wanfang. Our search will encompass studies published from the inception dates of these databases up to July 2023, and no language restrictions will be imposed during the search. Translation services and bilingual reviewers will be employed as needed. Two independent reviewers will be responsible for performing the search and meticulously screening all citations in accordance with predetermined search strategies. The detailed search strategies employed for the CENTRAL database can be found in Table 1, and these identical strategies will be applied to the other databases. Additionally, screening of the reference lists in the identified studies will be carried out to ensure that no relevant studies were inadvertently overlooked during the initial database search. Table 1 provides an illustration of the selection process.

**Table 1:** Search strategy for CENTRAL database.

Number	Search terms
1	Mesh descriptor: (Immunologic Conjunctivitis*) explode all trees
2	((Immunologic Conjunctivitis*) or (Vernal keratoconjunctivitis*) or (VKC*) or (Allergic Conjunctivitis*) or (Spring Allergic Conjunctivitis*) or (SAC*) or (Conjunctivitis*)):tl, ab, kw
3	Or:1–2
4	Mesh descriptor: (NLRP3*) explode all trees
5	((NLRP3*) or (NLRP3 inflammasome*) or (NLRP3 inflammasome inhibitors) or (IL-1β*) or (Interleukin-1 beta*) or (IL-18*) or (Interleukin-18*) or (Caspase-1*)):tl,ab,kw
6	Or:4–5
7	Mesh descriptor: (randomized controlled trial)explode all trees
8	((clinical study*) or (clinical trial*) or (controlled clinical trial*) or (randomized controlled trial*) or (RCT*) or (random*) or (randomly*) or (trial*)): to,ab,kw
9	Or 7–8
10	3 and 6 and 9

### Data extraction

#### Selection of studies

**Fig 1:**
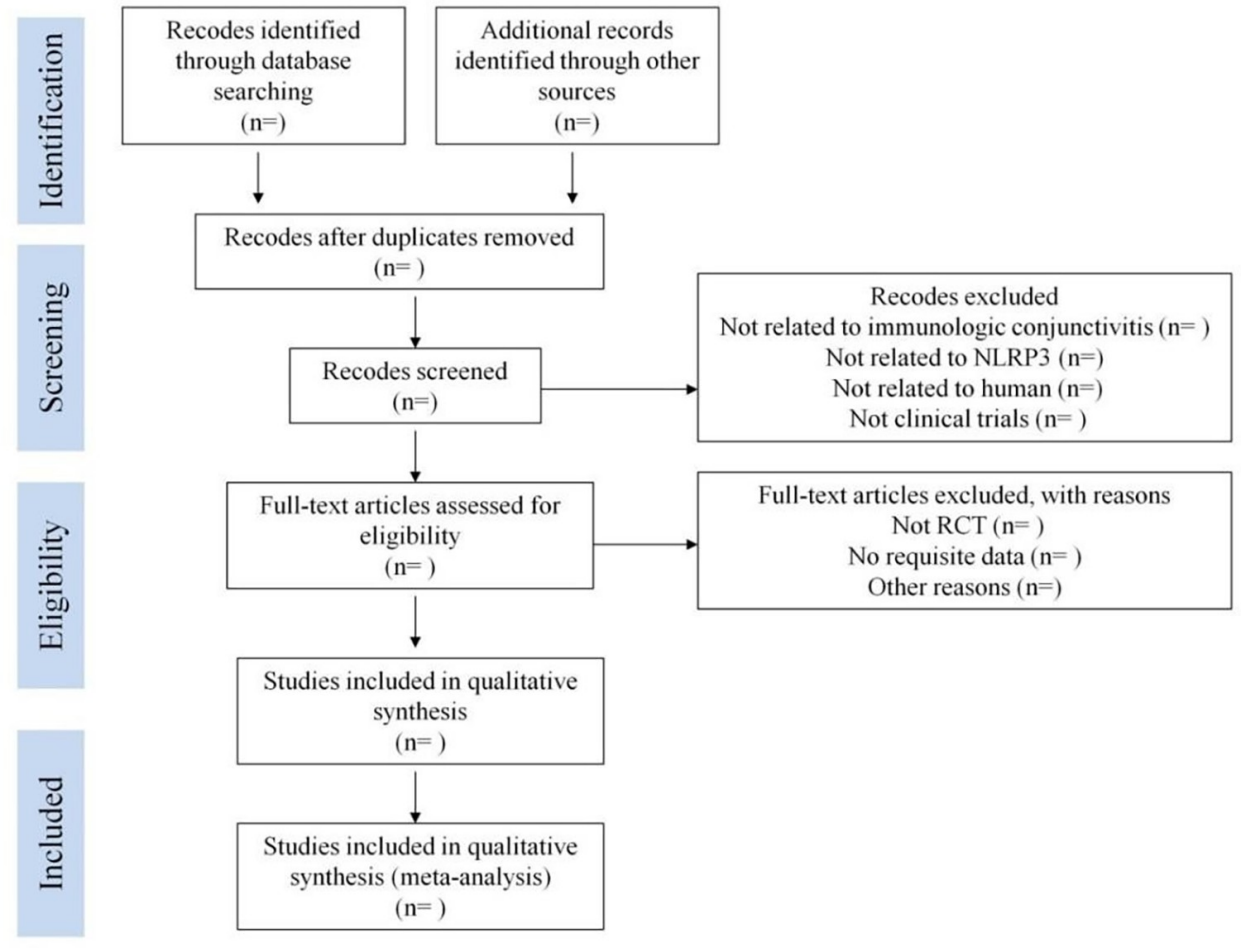
The flow chart of study selection process. RCT = randomized controlled trial.

Two independent reviewers will conduct an initial screening to identify potentially eligible studies. The retrieved literature will then be imported into literature management software, such as EndNote X9, to facilitate the removal of duplicate studies. Subsequently, the two reviewers will independently evaluate the titles and abstracts of the remaining studies based on the predefined inclusion and exclusion criteria, removing any irrelevant studies. Finally, the full-text articles of the remaining studies will be thoroughly read and assessed for eligibility. In the case of any disagreements, discussions will be held between the reviewers. If a consensus cannot be reached, a third reviewer with expertise in the field of immunology and conjunctivitis research will be consulted to resolve the issue, ensuring an unbiased assessment. The selection process will be presented in a flowchart, adhering to the PRISMA guidelines. Fig 1 provides an illustration of the selection process.

#### Data extraction and management

Two investigators will independently perform data extraction and complete pre-designed forms specific to this study. In the event of any disagreements, a third investigator will be consulted to resolve them. The following specific information will be included in the data extraction process: first author, publication date, study location, study setting, diagnostic criteria, eligibility criteria, funding sources, conflicts of interest, and demographic information such as age, sex, and race, as well as the number of patients in each group. Additional details will be collected regarding the randomization method, concealment, blinding, intervention details including medication, dosage, frequency, and duration, and all outcome measures and adverse events. To provide a comprehensive overview, we will also include a table summarizing the excluded studies and the reasons for their exclusion.

#### Risk of bias assessment

The risk of bias in all the included randomized controlled trials (RCTs) will be evaluated by two reviewers utilizing the RoB 2 Version 2 of the Cochrane risk-of-bias tool for randomized trials. This updated tool for assessing bias risk in randomized trials encompasses multiple domains, including random sequence generation (selection bias), allocation concealment (selection bias), blinding of participants and personnel (performance bias), blinding of outcome assessment (detection bias), incomplete outcome data (attrition bias), selective outcome reporting (reporting bias), and potential sources of other biases. It offers a systematic framework for appraising the internal validity of individual RCTs, aiding reviewers and researchers in making informed judgments regarding the dependability of trial outcomes [26].

To report the results of the bias risk assessment, we will generate either a summary table or a risk of bias graph. Each criterion will be categorized as "Low risk," "High risk," or "Unclear risk" [26]. In cases of discrepancies between the reviewers, they will be resolved through discussion. If a consensus cannot be reached, a third reviewer will be consulted for arbitration. In the context of a systematic review using RoB 2 (the Cochrane risk-of-bias tool); studies with a high risk of bias will be assessed separately in sensitivity analyses to evaluate their potential impact on the overall findings.

#### Treatment effect measurement

For dichotomous variables, the rate ratio (RR) will be reported. For continuous variables, either the mean difference (MD) or standardized mean difference (SMD) will be presented. Confidence intervals (CIs) of 95% will be applied to both dichotomous and continuous variables.

#### Dealing with missing data

In cases where the required data are insufficient, missing, or unclear, we will make efforts to contact the corresponding authors via email. If the authors cannot be reached or are unable to provide the missing data, we will analyze the available data and acknowledge this limitation in our discussion.

#### Assessment of heterogeneity

Statistical heterogeneity among the included studies will be assessed using the χ^2^ test and quantified with I^2^ values. The choice to employ a fixed or random-effects model should not only on the results of a statistical test but also depend on whether the studies exhibit a common effect size. While a *P*-value greater than 0.1 and an I^2^ value less than 50% may suggest no significant heterogeneity, and would favor the use of a fixed-effect model for the meta-analysis, but the test for heterogeneity can have limited power. The decision to use a random-effects model will consider not only the statistical thresholds but also the clinical and methodological differences among the studies. The I^2^ and χ^2^ thresholds are not definitive rules but general guidelines. Additionally, sensitivity analysis and subgroup analysis will be conducted to explore potential reasons for heterogeneity and provide a more nuanced understanding of the study results.

#### Data synthesis and analysis

Data analysis will be performed using Review Manager 5.4.1 software from the Cochrane Collaboration. We will select either a random-effects model or a fixed-effects model for data synthesis based on the results of the heterogeneity test. If the I^2^ value is less than 50%, indicating low heterogeneity, the fixed-effects model will be used. Conversely, if the heterogeneity is significant with an I^2^ value of 50% or higher, a random-effects model will be employed. For dichotomous data, the Mantel-Haenszel (M-H) method will be utilized to calculate the rate ratios (RRs) with a 95% confidence interval (CI). For continuous data, the inverse variance (IV) method will be applied to calculate the mean difference (MD) with a 95% CI.

#### Subgroup analysis

If substantial heterogeneity is observed and the required data are available, subgroup analyses will be conducted based on NLRP3-related factors, such as Interleukin-1 beta, Interleukin-18, or Caspase-1.

#### Sensitivity analysis

Sensitivity analyses will be conducted to assess the robustness of key decisions made during the review process and to evaluate the stability of our results. These analyses will involve examining the impact of factors such as sample size, study quality, methodological considerations, and missing data on the overall findings. For instance, sample size will be assessed by excluding or weighting studies with small sample sizes. Study quality will be evaluated by excluding studies with a high risk of bias. Methodological considerations will involve exploring the impact of different inclusion/exclusion criteria or data analysis methods. Missing data on the overall findings can be assessed by employing imputation methods or conducting sensitivity analyses to evaluate its potential impact. These detailed assessments will provide a comprehensive understanding of how variations in these factors might affect the overall conclusions of the study.

#### Publication bias assessment

If the number of included studies is at least 10, a funnel plot analysis will be conducted to assess publication bias [27]. In case visual examination of the funnel plot indicates asymmetry, we will explore potential sources of bias and consider adjusting for publication bias. This adjustment may involve using techniques such as trim and fill, or applying statistical methods like the Egger’s test for further investigation [28].

#### Evidence quality evaluation

The Grading of Recommendations Assessment, Development, and Evaluation (GRADE) system will be employed to assess the quality of evidence [29]. The GRADE system evaluates the quality of evidence based on several key components, including study design, risk of bias, consistency, directness, and precision. These domains contribute to the overall quality rating of the evidence. The quality of evidence will be categorized as "high," "moderate," "low," or "very low" based on the following criteria:

High Quality: The evidence is derived from well-conducted studies with consistent results and a low risk of bias. It provides a high degree of confidence in the findings.

Moderate Quality: The evidence is based on studies with some limitations but still provides a reasonable level of confidence in the results.

Low Quality: The evidence has significant limitations, including methodological flaws or inconsistency in results, leading to reduced confidence in the findings.

Very Low Quality: The evidence is characterized by serious limitations, high risk of bias, inconsistency, or imprecision. Confidence in the results is very limited.

It’s important to note that the GRADE system acknowledges potential subjectivity and biases in the process of evidence quality evaluation. Efforts will be made to minimize these limitations by employing a systematic and transparent approach to evidence assessment.

## Discussion

Although several studies have demonstrated the efficacy and safety of NLRP3-related treatments for immunologic conjunctivitis, a comprehensive systematic evaluation has not yet been conducted. This study represents the first systematic review and meta-analysis protocol aiming to assess the effectiveness and safety of NLRP3-related treatments for immunologic conjunctivitis. The protocol includes a comprehensive search across various databases, without any language restrictions. The results of this study will provide an updated and detailed summary of the available evidence concerning NLRP3-related treatments for patients with immunologic conjunctivitis. This evidence will be valuable for clinical practitioners and health policymakers in guiding the specific utilization of NLRP3-related treatments for patients with immunologic conjunctivitis. It is important to note that due to the limited number and quality of existing randomized controlled trials (RCTs) on NLRP3-related treatments for immunologic conjunctivitis, further high-quality RCTs are needed to strengthen the clinical evidence supporting NLRP3-related treatments as potential options for treating immunologic conjunctivitis.

## Supporting information

S1 Checklist PRISMA 2020 checklists*. (DOC)

## References

[1] Ono SJ. Molecular genetics of allergic diseases. Annu Rev Immunol. 2000;18:347–66. doi: 10.1146/annurev.immunol.18.1.347 . 10837062

[2] Bielory L, Delgado L, Katelaris CH, Leonardi A, Rosario N, Vichyanoud P. ICON: Diagnosis and management of allergic conjunctivitis[J].Ann Allergy Asthma Immunol, 2020,124(2):118–134. 31759180 10.1016/j.anai.2019.11.014

[3] Sompayrac Lauren. How The Immune System Works [M].6th edition:Wiley-Blackwell press,2019:111.

[4] Kenny SE, Tye CB, Johnson DA, Kheirkhah A. Giant papillary conjunctivitis: A review[J]. Ocul Surf,2020,18(3):396–402. 32339665 10.1016/j.jtos.2020.03.007

[5] Lee Feifei, Wanhong liao. Understanding and Treatment of Allergic Conjunctivitis in Traditional Chinese and Western Medicine(Chinese), 2019:28(21): 2379–2383

[6] Ge Jian, Wang Ningli. Ophthalmology [M] (Chinese). The Third Edition. Beijing: People’s Health Outreach, 2016:177

[7] Elieh Ali Komi D, Rambasek T, Bielory L. Clinical implications of mast cell involvement in allergic conjunctivitis. Allergy. 2018 Mar;73(3):528–539. doi: 10.1111/all.13334 Epub 2017 Nov 20. . 29105783

[8] Castillo M, Scott NW, Mustafa MZ, Mustafa MS, Azuara-Blanco A. Topical antihistamines and mast cell stabilisers for treating seasonal and perennial allergic conjunctivitis. Cochrane Database Syst Rev. 2015 Jun 1;(6):CD009566. doi: 10.1002/14651858.CD009566.pub2 . 26028608 PMC10616535

[9] Erlich Z, Shlomovitz I, EImmunologic conjunctivitisy-Botzer L, Cohen H, Frank D, Wang H, et al. Macrophages, rather than DCs, are responsible for inflammasome activity in the GM-CSF BMDC model[J]. Nat Immunol,2019,20(4):397–406. 30742078 10.1038/s41590-019-0313-5

[10] Hochheiser IV, Pilsl M, Hagelueken G, Moecking J, Marleaux M, Brinkschulte R, et al. Structure of the NLRP3 decamer bound to the cytokine release inhibitor CRID3[J]. Nature,2022,604(7904):184–189. 35114687 10.1038/s41586-022-04467-w

[11] Orecchioni M, Kobiyama K, Winkels H, Ghosheh Y, McArdle S, Mikulski Z, et al. Olfactory receptor 2 in vascular macrophages Immunologic conjunctivitisives atherosclerosis by NLRP3-dependent IL-1 production[J].Science,2022,375(6577):214–221. 35025664 10.1126/science.abg3067 PMC9744443

[12] Chen Y, Lai L, Mo Z, Li X, Su X, Li Y, et al. Mulberry Leaf Extract Alleviates Staphylococcus aureus-Induced Conjunctivitis in Rabbits via Downregulation of NLRP3 Inflammasome and Upregulation of the Nrf2 System and Suppression of Pro-Inflammatory Cytokines. Pharmacology. 2022;107(5–6):250–262. doi: 10.1159/000523786 Epub 2022 Apr 13. . 35417907

[13] Yamada J. [Alleviation of seasonal allergic symptoms with superfine beta-1,3-glucan: a randomized study]. Nippon Ganka Gakkai Zasshi. 2009 Nov;113(11):1082–7. Japanese. . 19994586

[14] Leonardi A, Daull P, Garrigue JS, Cavarzeran F, Docquier M, Di Stefano A, et al. Conjunctival transcriptome analysis reveals the overexpression of multiple pattern recognition receptors in vernal keratoconjunctivitis. Ocul Surf. 2021 Jan;19:241–248. doi: 10.1016/j.jtos.2020.09.009 Epub 2020 Oct 21. . 33098984

[15] Leonardi A, Curnow SJ, Zhan H, Calder VL. Multiple cytokines in human tear specimens in seasonal and chronic allergic eye disease and in conjunctival fibroblast cultures. Clin Exp Allergy. 2006 Jun;36(6):777–84. doi: 10.1111/j.1365-2222.2006.02499.x . 16776679

[16] Takano H, Osakabe N, Sanbongi C, Yanagisawa R, Inoue K, Yasuda A, et al. Extract of Perilla frutescens enriched for rosmarinic acid, a polyphenolic phytochemical, inhibits seasonal allergic rhinoconjunctivitis in humans. Exp Biol Med (Maywood). 2004 Mar;229(3):247–54. doi: 10.1177/153537020422900305 . 14988517

[17] Leonardi A, Borghesan F, DePaoli M, Plebani M, Secchi AG. Procollagens and inflammatory cytokine concentrations in tarsal and limbal vernal keratoconjunctivitis. Exp Eye Res. 1998 Jul;67(1):105–12. doi: 10.1006/exer.1998.0499 . 9702183

[18] Leonardi A, Tarricone E, Corrao S, Alaibac M, Corso AJ, Zavan B, et al. Chaperone patterns in vernal keratoconjunctivitis are distinctive of cell and Hsp type and are modified by inflammatory stimuli. Allergy. 2016 Mar;71(3):403–11. doi: 10.1111/all.12814 Epub 2015 Dec 23. . 26613380

[19] Micera A, Di Zazzo A, Esposito G, Sgrulletta R, Calder VL, Bonini S. Quiescent and Active Tear Protein Profiles to Predict Vernal Keratoconjunctivitis Reactivation. Biomed Res Int. 2016;2016:9672082. doi: 10.1155/2016/9672082 Epub 2016 Feb 17. ; PMCID: PMC4773530. 26989694 PMC4773530

[20] Page MJ, McKenzie JE, Bossuyt PM, Boutron I, Hoffmann TC, Mulrow CD, et al. The PRISMA 2020 statement: an updated guideline for reporting systematic reviews. BMJ 2021;372:n71. doi: 10.1136/bmj.n71 33782057 PMC8005924

[21] Chinese Medical Association Ophthalmological Society Cornea Group. Expert Consensus on Diagnosis and Treatment of Allergic Conjunctivitis in China (2018) [J]. Chinese Journal of Ophthalmology, 2018, 54(6): 409–414.

[22] American Academy of Ophthalmology Cornea /External Disease Panel. Preferred Practice Pattern@ Guideline. Conjunctivitis. [R/OL] (2013)[2018-06-25]. http://www.aao.org/ppp.

[23] Ge J., & Wang N. (2016). Ophthalmology (3rd ed.). Beijing: People’s Medical Publishing House. (p. 177).

[24] Pullerits T, Praks L, Skoogh BE, Ani R, Lotvall J. Randomized placebo-controlled study comparing a leukotriene receptor antagonist and a nasal glucocorticoid in seasonal allergic rhinitis. Am J Respir Crit Care Med 159:1814–1818, 1999. doi: 10.1164/ajrccm.159.6.9810016 10351924

[25] Howarth PH, Stem MA, Roi L, Reynolds R, Bousquet J. Double-blind, placebo-controlled study comparing the efficacy and safety offexofenadine hydrochloride (120 and 180 mg once daily) and cetirizine in seasonal allergic rhinitis. J Allergy Clin ImmunolI04:927–933, 1999. doi: 10.1016/s0091-6749(99)70070-9 10550734

[26] Higgins JPT, Savović J, Page MJ, et al. Revised Cochrane risk of bias tool for randomized trials (RoB 2)(22 August 2019). Available at: https://www.riskofbias.info/welcome/rob-2-0-tool/currentversion-of-rob-2.

[27] Peters JL, Sutton AJ, IMMUNOLOGIC CONJUNCTIVITIS Jones, Abrams KR, Rushton L. Contour-enhanced meta-analysis funnel plots help distinguish publication bias from other causes of asymmetry. Journal of clinical epidemiology. 2008; 61(10):991–6. Epub 2008/06/10. doi: 10.1016/j.jclinepi.2007.11.010 18538991

[28] Egger M, Davey Smith G, Schneider M, Minder C. Bias in meta-analysis detected by a simple, graphical test. BMJ (Clinical research ed). 1997; 315(7109):629–34. Epub 1997/10/06. doi: 10.1136/bmj.315.7109.629 9310563 PMC2127453

[29] Guyatt GH, Oxman AD, Vist GE, Kunz R, Falck-Ytter Y, Alonso-Coello P, et al. GRADE: an emerging consensus on rating quality of evidence and strength of recommendations. BMJ (Clinical research ed). 2008; 336(7650):924–6. Epub 2008/04/26. doi: 10.1136/bmj.39489.470347.AD . 18436948 PMC2335261

